# Vinyl as Fine Wine: The Role of Expectation on the Perception of Music Format

**DOI:** 10.3389/fpsyg.2022.873517

**Published:** 2022-05-26

**Authors:** Rickard Enstroem, Rodney Schmaltz

**Affiliations:** ^1^Department of Decision Sciences, School of Business, MacEwan University, Edmonton, AB, Canada; ^2^Department of Psychology, Faculty of Arts and Science, MacEwan University, Edmonton, AB, Canada

**Keywords:** expectation, music, vinyl records, digital music, sound quality

## Abstract

While vinyl, compact discs, and even eight-track tapes were traditionally promoted to consumers as producing superior sound, the introduction of compressed digital music, such as mp3s, was markedly different. Initially, one of the primary selling features of digital music was convenience and portability rather than sound quality. Recently, vinyl music sales have experienced a substantial resurgence. Waveforms from vinyl represent recorded music more accurately than compressed digital formats and have the potential to produce better sound. Even so, most music listeners do not reliably listen to music on audiophile quality high-end equipment. For this reason, we believe one aspect of vinyl sales is the *expectation* that vinyl quality is superior. In this study, we sought to isolate the contribution of expectation to perceived sound quality. Participants were asked to listen to a selection of music on either vinyl or mp3. Some participants were told that they were listening to vinyl when the musical selection was an mp3, while others were told they were listening to an mp3 while actually listening to vinyl. A multivariate analysis through a Canonical Correlation Analysis established that expectation of music format quality drove post-listening evaluations.

## Introduction

While overall global sales of physical copies of music, such as compact discs, are declining, the one category of physical sales that has continued to increase over the past several years is vinyl records (Friedlander, 2018, 2020; Hoose, 2018; MRC Data, 2021). Preference for vinyl has multidimensional bases, spanning such as sonic qualities (Yochim and Biddinger, 2008; Bartmanski and Woodward, 2015; Brown and Krause, 2020), an extension of self through the symbolism of eclecticism (Brown and Krause, 2020), nostalgia (Yochim and Biddinger, 2008; Brown and Krause, 2020), and authenticity (Yochim and Biddinger, 2008; Bartmanski and Woodward, 2015; Brown and Krause, 2020). A unifying movement across generations of music format users, be it Edison cylinders, 78s shellac records, vinyl records, or cassettes, is also the emergence of subcultures at the end of eras, bemoaning technology progress by holding on to past formats (Plasketes, 1992). Like many other products signalling eclectism, status, and novelty, vinyl records are also susceptible to prestige-seeking consumer behaviour (Vigneron and Johnson, 1999).

Expectation plays a role in how we interpret our physical and social world. It influences perceptions of interpersonal power (Baldwin et al., 2009), how teachers view students (Rosenthal and Jacobson, 1968), and in medicine, expectation relates to placebo and nocebo effects (e.g., De la Fuente-Fernández et al., 2001; Geuter et al., 2017). Other pertinent examples of the influence of expectation are the extent to how much people enjoy a visit to a museum (Pekarik and Schreiber, 2012), how they interpret song lyrics (Fried, 1996; North and Hargreaves, 2005), and even how they experience the taste of food. For example, the type of cup used to serve coffee directly impacts both the expectation of coffee flavour and the actual ratings of enjoyment of the coffee (Carvalho and Spence, 2019). Vinyl records are often viewed as sounding richer (Yochim and Biddinger, 2008; Byrne, 2012; Brown and Krause, 2020) and warmer (Yochim and Biddinger, 2008; Brown and Krause, 2020) than other music formats. The explanation is that compressed files, such as mp3, employ lossy coding where the digital file is changed in ways that may potentially change the listening experience (e.g., Pohlmann, 2011). However, how the record is mastered, the vinyl weight and the quality and setup of the record player and amp also directly impact the listening experience. Despite this, there is a pronounced expectation that vinyl sounds better than other music formats. A suitable analogy for vinyl appreciation may be found in consumer evaluations of wine quality. For instance, when people are *told* that wine is expensive, it is rated as more appealing (Plassmann et al., 2008). There is also increased activity in the brain’s medial orbitofrontal cortex, a brain area associated with pleasurable experiences. However, this effect does not occur when people drink the same wine under the guise that it is inexpensive.

Interestingly, the actual price of wine is not a reliable indicator of enjoyment. In a large sample of blind tastings, researchers found a *negative* correlation between wine price and enjoyment ratings, but only for people who do not have wine training (Goldstein et al., 2008). The implication is that when an average person does not know the wine price, more expensive wines tend to be rated slightly lower than less expensive wines. Instead, experts and experienced wine tasters exhibited the opposite effect; a positive correlation between the wine’s price and rating.

Based on the previous research on expectation, it is reasonable to assert that expectation is one aspect of perceived sound quality for the average consumer. As vinyl may contain a more accurate representation of recorded music, experts—be they producers, professional musicians, or audiophiles—would be able to detect the difference between vinyl and compressed music. Nonetheless, most music consumers would not be classified as audiophiles, with some estimates indicating as few as 150,000 audiophiles worldwide (Del Colliano, 2019). Therefore, this research focuses on the average music consumer, not audiophiles. The purpose of this research is not to determine if the sound of vinyl is superior to digital formats or vice-versa; instead, we are interested in the impact of *expectation* on the listening experience.

### Purpose of the Study

Previous research has explored the impact of music format, specifically vinyl or CD, on emotional arousal and nostalgia (Lepa and Tritakis, 2016). Participants were asked to listen to a song and provided with the information that it would play from either a CD or a vinyl record. The researchers then manipulated the format played; some participants were correctly told that they were listening to vinyl, while others listened to music from a CD under the guise of vinyl. Regardless of the format told, participants reported a stronger emotional reaction when the music was played from a vinyl record rather than a CD.

In exploring the role of expectation in perceived sound quality, participants were randomly assigned to one of six conditions: expect to listen to vinyl and hear vinyl (ExpVINYL–PlayVINYL); expect to listen to vinyl and hear MP3 (ExpVINYL–PlayMP3), expect to listen to MP3 and hear MP3 (ExpMP3–PlayMP3); expect to listen to MP3 and hear vinyl (ExpMP3–PlayVINYL); and two control conditions where participants were not told anything and heard either vinyl (VINYL) or mp3 (MP3). All participants were made aware that the mp3 was encoded at 192 kbps, a medium-quality mp3 bitrate. Music from streaming services was not included in avoiding biases regarding preferences for different streaming services and removing ambiguity about the quality of the music format. As different streaming services provide a range of audio quality, we used mp3 as a variable and clearly stated the bitrate at which the mp3 was encoded.

## Materials and Methods

### Sample

In total, 176 participants (115 females, 61 males) were randomly selected from the research participation pool in the Department of Psychology who received partial research credit toward an introductory psychology course requirement. As age was not a variable of interest, we asked participants to select their age group. The groups were classified as: (a) under 18 years of age; (b) 18–20 years of age; (c) 21–23 years of age; (d) 24–30 years of age; or (e) 30+ years of age. 63% of participants were between the ages of 18 and 20, 24% were between 21 and 23, and the remaining 13% were distributed across the other age categories. All participants provided written consent prior to their participation, and the University’s Research Ethics Board approved the study.

### Design

Participants were brought to a lab in groups of up to three. All participants were seated as a group in a room with stereo equipment, a laptop with iTunes visible on the screen, a record player, and an AV receiver. The stereo equipment included a Marantz PN-5003 amplifier and a Pro-Ject Debut turntable. A copy of Frank Sinatra’s *Come Fly With Me* was displayed against the record player such that it was visible to participants. This record was mastered for vinyl (for an overview of the limitations of vinyl recordings and the requirements for creating ideal-sounding vinyl recordings, see Hoose, 2018).

To conceal the hypothesis, the experimenter provided participants with a cover story that the purpose of the study was to evaluate different music styles and understand if different personality characteristics impacted the perception of different musical pieces. Following this, participants were led into individual rooms which contained headphones (Panasonic RP-HT455) and a copy of the dependent measures, which included questions on perceived sound quality. The headphones were connected to the receiver in the main room *via* a powered splitter to ensure that there would not be a loss in sound quality. Participants were not able to see one another in these rooms. The experimenter then went back to the main room to check which condition the participants were assigned.

Upon learning the experimental condition, the experimenter was positioned in the lab so that the participants could hear the experimenter but not see the experimenter. This setup was chosen to minimise interaction with participants after the experimenter was unblinded to the condition. In all conditions, the experimenter then informed participants that the music was about to begin and that they should put on their headphones.

Participants listened to the Frank Sinatra song, *Come Fly With Me*,^1^ and either heard a vinyl version of the song or a 192 kbps mp3. Following the music selection, participants completed the dependent measures of sound quality. The measures included a manipulation check where participants were asked to report which music format was played. Following this, participants who did not recall the experimenter-told format were removed from the data set. Participants were also asked to complete a suspiciousness probe, with the data subsequently removed for participants who indicated that they were aware of the hypothesis.

Participants were asked to rate the song’s overall sound quality, along with the specific dimensions of clarity, smoothness, fullness, and distinctness. In rating the quality dimensions, the participants responded on a 9-point Likert scale. In this study, we were interested in the subjective experience of sound quality. For our dependent measures, we wanted terms that would be understandable and relatable to a student population. To develop these terms, we conducted a brief survey of students and asked them to listen to a piece of music on mp3 and vinyl. We then asked the students to list all terms they could think of to describe the sound quality of the music. From this list, we took the most commonly used terms across all music formats and used these as our dependent measures. To ensure that participants in the current study understood these terms, we added a brief explanation of each term based on a dictionary definition. In our measures, we collapsed some terms that were close in definition (i.e., warmth and richness were collapsed under fullness).

Consequently, “overall” was defined as the “overall impression of the song’s sound quality,” whereas “distinctness” alluded to the design of stereo recordings “so that the different aspects of the music sound like they are coming from different directions all around you.” In defining “clarity,” it was stated as “how clear and crisp the sound was,” with “a minimum of background noise.” “Smoothness,” in turn, indicated the extent to “how smooth the playback was,” if it was “choppy,” or did “lag at parts.” Last, “fullness” was defined as “how rich and warm the playback sounds.”

## Results

**FIGURE 1:**
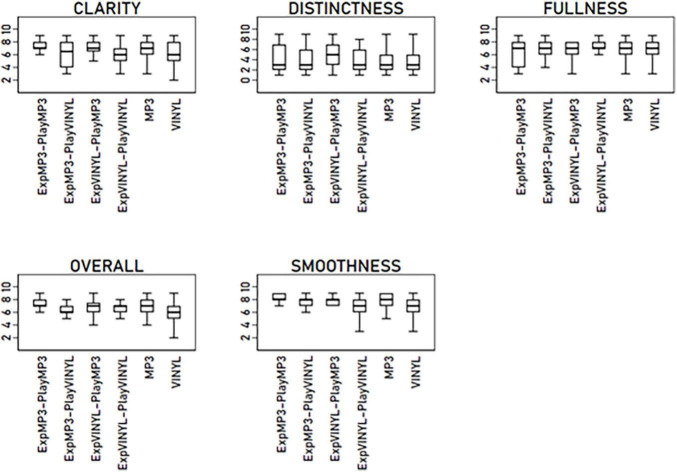
Boxplots of the dependent measures across the experimental conditions.

Figure 1 presents boxplots of the six dependent sound quality measures across the experimental conditions. Examining the boxplots reveals a fair bit of separation in terms of medians among the experimental conditions within each dependent measure. The exception is FULLNESS, where the medians are more or less aligned. The two base conditions, MP3 and VINYL, are primarily separated in CLARITY, OVERALL, and SMOOTHNESS.

As shown, the dispersion varies both within and across the dependent measures in terms of the interquartile ranges. Most agreement in interquartile dispersions across experimental conditions is demonstrated for OVERALL and SMOOTHNESS; these dependent measures also exhibit the most compressed dispersions. The boxplots illustrate that all dependent measures exhibit a combination of symmetric and positively and negatively skewed distributions across the experimental conditions.

The multivariate relationship between the six experimental conditions and the five sound quality dimensions was analysed through a Canonical Correlation Analysis (CCA).^2^ The analytical approach and modelling design in this study follow the design previously recommended by Enstroem and Schmaltz (2017) when exploring the multivariate relationship between music immersion through the five-factor MUSIC model of musical preferences (Rentfrow et al., 2011) and risk-taking likelihood, as measured by the DOSPERT scale (Blais and Weber, 2006).

**FIGURE 2:**
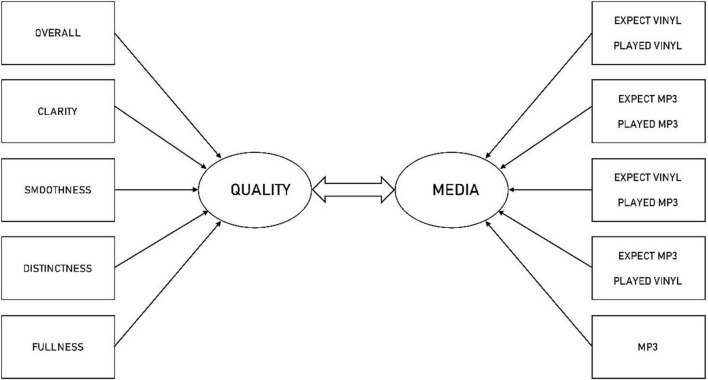
Canonical Correlation model of the relationship between the experimental conditions and the dependent sound quality measures.

Figure 2 presents the canonical correlation model of the relationship between the variable sets representing perceived SOUND QUALITY and the experimental conditions representing MEDIA. In the analysis, VINYL served as the control group, and the five dependent measures were participants’ perception of OVERALL, CLARITY, SMOOTHNESS, DISTINCTNESS, and FULLNESS. All variables were mean-centred before the analysis, and the estimated coefficients presented here are standardised coefficients.

The analysis resulted in five canonical functions with squared canonical correlations of 0.21, 0.043, 0.034, 0.0086, and 0.00000179. Jointly, the full model across all five functions was statistically significant according to Wilks’ λ criterion: λ = 0.727907, *F*_(25, 618.165)_ = 2.2069, *p* < 0.0007. Thus, as Wilks’ λ represents the variance unexplained by the model (1 − λ) yields the full model effect size as an *R*^2^ metric. The implication is that the full model explains nearly 30% of the shared variance between the two variable sets representing MEDIA, on the one hand, and SOUND QUALITY, on the other hand.

In assessing the dimensionality of expectation’s impact on sound quality, the results show that the full model, comprised of Canonical Functions 1–5, was statistically significant, but the Canonical Functions 2–5 were insignificant. Furthermore, given the magnitude of the squared canonical functions, only the first canonical function was deemed noteworthy in this study, with a 27.2% shared variance. Therefore, the impact of media expectation on perceived quality is mapped out and interpreted as a one-dimensional solution in the canonical correlation analysis.

Table 1 presents the standardised canonical function coefficients for Canonical Function 1. The estimated standardised coefficients show that the relevant criterion variables are CLARITY, SMOOTHNESS, and FULLNESS. In the predictor variable set, treatments ExpMP3–PlayMP3, ExpVINYL–PlayMP3, and MP3 were the primary contributors to the synthetic predictor variable MEDIA.

**TABLE 1:** Canonical solution for sound quality and experimental conditions.

Variable	Standardised coefficient
**Dependent measures**	
OVERALL	–0.1699
CLARITY	0.7096
SMOOTHNESS	0.5763
DISTINCTNESS	0.0505
FULLNESS	–0.4403
**Experimental conditions**	
ExpVINYL–PlayVINYL	–0.2123
ExpMP3–PlayMP3	0.8111
ExpVINYL–PlayMP3	0.5294
ExpMP3–PlayVINYL	0.1402
MP3	0.6249

The attribute FULLNESS arguably captures the essence of the vinyl sound. Correspondingly, in comparison to vinyl, MP3 is negatively related to the perceived fullness of the sound. Furthermore, considering the impact of expectation, the condition ExpMP3–PlayMP3 is even stronger negatively related to FULLNESS. This result points to a negative expectation effect of MP3 sound regarding FULLNESS in comparison to vinyl. Conversely, when hearing an mp3 sound being played but told that it is vinyl, as in the ExpVINYL–PlayMP3 condition, it results in a weaker negative correlation with FULLNESS than in the MP3 and ExpMP3–PlayMP3 conditions; consequently, this result demonstrates the positive expectation effect of vinyl sound in relationship to FULLNESS.

CLARITY and SMOOTHNESS are both connected to the digital qualities of sound. For SMOOTHNESS, this finding may seem unforeseen as the labelling suggests distinct vinyl sound qualities. However, in the survey, SMOOTHNESS alluded to the sound quality by inquiring “was the playback choppy and did it lag at parts or was the playback very smooth,” which illustrates the benefits of the digital sound. The findings thus far demonstrate that the MP3 treatment condition, where participants heard the song playing through an MP3, is positively correlated with the digital sound qualities of CLARITY and SMOOTHNESS in comparison to the VINYL control group. This result is a core result in that, on average, participants were able to distinguish in straight untold listening the difference in sound quality between the medium-quality mp3 bitrate and vinyl music.

In limning the expectation effect of MP3, the ExpMP3–PlayMP3 condition exhibits an even stronger positive correlation with CLARITY and SMOOTHNESS than the MP3 condition, in comparison to VINYL. Therefore, this result captures a positive expectation effect in that the digital sound expectation reinforces the perceived digital sound qualities related to CLARITY and SMOOTHNESS.

The expectation effect of vinyl upon mp3 is estimated through the impact of the ExpVINYL–PlayMP3 condition. Compared to the ExpMP3– PlayMP3 condition, it has a weaker relationship with the sound qualities CLARITY and SMOOTHNESS; therefore, this finding suggests a negative expectation effect in that the expectation of vinyl sound attenuates the digital sound qualities.

**FIGURE 3:**
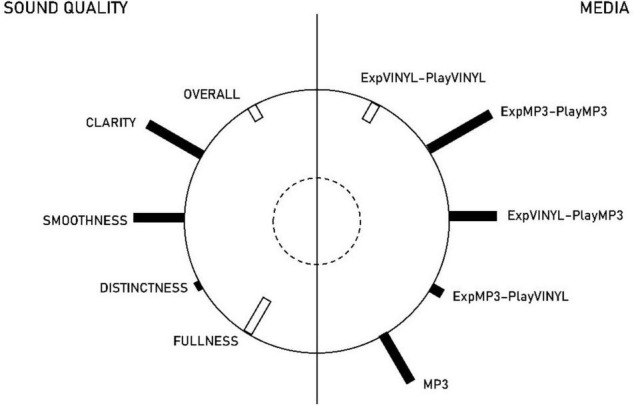
Heliograph showing the estimated multivariate relationship between media and sound quality.

Figure 3 presents a heliograph as a visual illustration of the analysis. The lengths of the bars indicate the magnitudes of the standardised coefficients, as presented in Table 1. Furthermore, the black-coloured bars represent standardised coefficients with a positive sign, whereas the white-coloured bars indicate standardised coefficients with negative signs. On the right side of Figure 3, the experimental conditions representing MEDIA are represented, with the dependent measures representing SOUND QUALITY to the left. Thus, Figure 3 shows that the synthetic predictor variable MEDIA was primarily contributed to by the ExpMP3–PlayMP3, ExpVINYL–PlayMP3, and MP3 experimental conditions. Similarly, the most impactful dependent measures for the synthetic dependent variable SOUND QUALITY were CLARITY, SMOOTHNESS, and FULLNESS.

The combinations of signs, as indicated by the colours of the bars, illustrate an inverse or positive relationship between the experimental conditions and the dependent measures. For example, it can be discerned that the MP3 is positively related to CLARITY, SMOOTHNESS, compared to VINYL, but negatively related to FULLNESS.

## Discussion

A multivariate analysis through a canonical correlation model demonstrated that expectation plays a pervasive and salient role in judging vinyl and mp3 recorded music formats. That is, the experience of listening to music is impacted by expectation and not due to sound quality alone. Our results can be interpreted through a Bayesian lens regarding the interplay between prior expectations and sensory evidence. Our results suggest that for vinyl, the expectations in terms of sound quality are so firmly established that participants rely at least partially on the prior expectations rather than the sensory evidence.

In our analysis, the three aspects of sound quality that emerged with prominent effect sizes were CLARITY, SMOOTHNESS, and FULLNESS. As defined in our study, CLARITY and SMOOTHNESS are strengths primarily associated with the digital sound, while FULLNESS is a distinct advantage of the vinyl recorded sound. Our estimations show how the respective expectations of vinyl and mp3 sound qualities operate on participants’ judgement by lessening and strengthening the perception of the underlying music format in the direction of vinyl vs. mp3-related sound qualities. Specifically, we found negative expectation effects of mp3 and vinyl upon the mp3-played format in that the expectation of mp3 made the perceived sound quality even lesser related to the vinyl quality of FULLNESS and that the expectation of vinyl resulted in a lessened relationship to the digital sound qualities of CLARITY and SMOOTHNESS. Similarly, we established positive expectation effects for vinyl and mp3 upon mp3 as the vinyl expectation resulted in a strengthened relationship with the vinyl quality of FULLNESS, and the mp3 expectation enhanced the association with the digital sound qualities of CLARITY and SMOOTHNESS.

These results should be conceived in the context that the participants could tease out the mp3-played song from the same song played on vinyl. Mp3s are a lossy music format, with the highest encoding possible at 320 kbps and the lowest at 32 kbps. As a comparison, A compact disc is encoded at 1,411 kbps. That the mp3 was encoded at the bitrate of 192 kbps makes our results even more compelling; even for this medium bitrate, and presumably wide gap in sound quality between vinyl and mp3, the expectation effect is found.

While it is true that analogue recordings may provide more accurate representations of recorded sound, the average music consumer does not necessarily have the audio equipment nor command the expertise to detect and gauge differences in nuances among the analogue and compressed digital formats. Despite this, there is a widespread belief that vinyl sounds better than other music formats. The takeaway from our results is that it is not the actual sound quality of vinyl alone that drives the preference, but rather the knowledge of vinyl’s better sound that impacts sound perception for the average music consumer. From a consumer judgement and decision-making standpoint and information processing dilemma, the knowledge of vinyl’s superior sound quality likely forms a salient heuristic that is easily accessible to the consumer and impacts the listening experience.

High-end equipment will most certainly produce a different listening experience between vinyl and compressed music formats. While the focus of this study was the average music consumer, a worthy extension is to explore the role of expectations on audiophiles. Based on previous research on expectation and wine tasting (Plassmann et al., 2008), we expect expectation to play a less salient role on audiophiles than the average music consumer. Beyond the person’s audiophile orientation, the results may differ depending on music interest and genre. In this exploratory work, we did not include contenders of lossless streaming music. Estimating the effect of brand expectation would yield additional insights into how expectations regarding factual vs. non-factual differences in sound quality operate.

## Conclusion

Music is an essential part of many people’s lives. This research showed that the experience of music quality is subjective and may be influenced by both expectation and sound quality. A multivariate analysis through a canonical correlation model revealed multiple positive and negative expectation effects that illustrate how expectations for both vinyl and mp3 sound qualities lessen or strengthen the perception of the played music format in the direction of vinyl- vs. mp3-related sound qualities. The commonly held belief that vinyl records produce superior sound may be driving the music listening experience. At least for the average music consumer, sound quality expectations play a role in the overall listening experience. Our results, showing that expectation plays a role in the perception of sound quality, is not meant to say that people should avoid vinyl records; instead, it is the opposite: if a person believes that vinyl sounds better, the listening experience will be better, regardless of sound quality.

## Data Availability Statement

The raw data supporting the conclusions of this article will be made available by the authors, without undue reservation.

## Ethics Statement

The studies involving human participants were reviewed and approved by MacEwan University Human Research Ethics Board. The participants provided their written informed consent to participate in this study.

## Author Contributions

RS designed the experiment. RE conducted the statistical modelling. Both authors contributed to the write-up of the manuscript and approved the submitted version.

## Conflict of Interest

The authors declare that the research was conducted in the absence of any commercial or financial relationships that could be construed as a potential conflict of interest.

## Publisher’s Note

All claims expressed in this article are solely those of the authors and do not necessarily represent those of their affiliated organizations, or those of the publisher, the editors and the reviewers. Any product that may be evaluated in this article, or claim that may be made by its manufacturer, is not guaranteed or endorsed by the publisher.

## Footnotes

1 To choose the right song for this study, we wanted to avoid any modern popular songs as participants may have strong opinions about the song. A local high-end audio equipment manager was consulted and asked which song was used to highlight equipment to consumers. Frank Sinatra’s *Come Fly With Me* was recommended.

2 For an overview of CCA, see Sherry and Henson (2005).
